# Effects of childhood obesity and related genetic factors on precocious puberty: protocol for a multi-center prospective cohort study

**DOI:** 10.1186/s12887-022-03350-x

**Published:** 2022-05-27

**Authors:** Tingting Yu, Ying Yu, Xiaoqing Li, Peng Xue, Xiaodan Yu, Yao Chen, Huijun Kong, Cuilan Lin, Xiumin Wang, Hao Mei, Dan Wang, Shijian Liu

**Affiliations:** 1 Sanya Women and Children’s Hospital, managed by Shanghai Children’s Medical Center, Shanghai Jiao Tong University School of Medicine, 339 Yingbin Road, Sanya, 572022 China; 2 Office of Hospital Infection Management, The Fourth Affiliated Hospital of Anhui Medical University, Hefei, Anhui China; 3 School of Public Health, Shanghai Jiao Tong University School of Medicine, Shanghai, China; 4 Department of Developmental and Behavioral Pediatrics, Shanghai Children’s Medical Center, Shanghai Jiao Tong University School of Medicine, Shanghai, China; 5 Department of Endocrinology and Genetic Metabolism, Shanghai Children’s Medical Center, Shanghai Jiao Tong University School of Medicine, Shanghai, China; 6 Department of Pediatrics, Qu Fu People’s Hospital, Qufu, Shandong China; 7 Boai Hospital of Zhongshan, Southern Medical University, Zhongshan, Guangdong China; 8 Department of Data Science, School of Population Health, University of Mississippi Medical Center, Jackson, MS USA; 9 Pediatric Translational Medicine Institute, Shanghai Children’s Medical Center, Shanghai Jiao Tong University School of Medicine, 1678 Dongfang Road, Shanghai, 200127 China

**Keywords:** Precocious puberty, Obesity, Cohort study, Single nucleotide polymorphism

## Abstract

**Background:**

Childhood obesity has important effects on the onset and development of puberty. Although a number of studies have confirmed the relationship between obesity and precocious puberty, little is known about the pleiotropic genes of obesity and precocious puberty and the interaction between genes and environment. There are four objectives: (1) to analyze the incidence of precocious puberty in the general population in China; (2) to verify the direct effect of obesity on children’s precocious puberty using a variety of methods; (3) to verify the effect of obesity and its risk gene polymorphism on precocious puberty in a prospective cohort study; and (4) to analyze the interaction effect of genes and environment on pubertal development.

**Methods:**

We will conduct a multi-center prospective cohort study in three cities, which are selected in southern, central, and northern China, respectively. Primary schools in these cities will be selected by a stratified cluster random sampling method. Primary school students from grade 1 to grade 3 (6 to 10 years old) will be selected for the cohort with extensive baseline data collection, including assessment of pubertal development, family demographic information, early development, sleep pattern, dietary pattern, and physical activity. Participants will be followed up for at least three years, and long-term follow-up will depend on future funding.

**Discussion:**

The findings of this multicenter prospective population-based cohort study may expand previous related puberty development research as well as provide important information on the mechanism of early puberty. Targeted interventions can also be developed to improve adolescent health problems related to puberty development based on the available evidence.

**Trial registration:**

ClinicalTrials.gov Identifier: NCT04113070, prospectively registered on October 2, 2019.

## Background

The high prevalence of childhood obesity has become a concerning public health problem. In 2016, over 340 million children and adolescents aged 5–19 globally were overweight or obese [1]. Moreover, an increasing number of countries are seeing earlier onset of puberty in children [2–4]. Early puberty has been reported to have a positive association with psychological symptoms, conduct problems, and reproductive diseases [5, 6]. Puberty is a critical but complex process, since many factors, including genetic [7–9], nutritional [10], and environmental factors [11], may influence its development.

Recently, growing evidence from cross-sectional [12–14], and longitudinal studies [15] has indicated that obesity or a higher body mass index (BMI) is associated with earlier timing of puberty in girls. However, the relationship between obesity and timing of puberty onset in boys is inconsistent. Although several studies reported that boys with higher BMI or obesity are more likely to experience early puberty [12, 15, 16], some studies have suggested that delayed puberty is associated with obesity [17, 18].

The occurrence of precocious puberty is closely related to obesity, and there exists some common pathogenesis. A previous study found a positive correlation between obesity and the level of leptin, a protein secreted by peripheral mature adipocytes; moreover, leptin and its receptor play key roles in the initiation of puberty onset and are important biomarkers involved in the regulation of both obesity and precocious puberty [19]. Genetically speaking, over 600 genetic locus are associated with obesity, and many studies have focused on the *LEPR*, *MC4R,* and *FTO* genes [20, 21]. In addition, precocious puberty is related to the *KISS1*, *MC4R*, and *LEPR* genes [7–9]. Consequently, pleiotropic genes like *LEPR* and *MC4R* could influence the phenotype of both obesity and precocious puberty; however, their effect on precocity puberty and its magnitude are still unknown.

Previous studies on the relationship between obesity and precocious puberty have mostly been cross-sectional studies, with few studies on pleiotropic genes of obesity and puberty. To explore the relationship between obesity and puberty development, this prospective cohort study will investigate the genetic, parental, social and family environment, lifestyle, and other determinants of puberty development. This protocol describes an overview of the design and methods of this cohort study, which is funded by the National Natural Science Foundation of China (grant number 81872637). The study objectives are as follows: I) analyzing the incidence of precocious puberty in the general population in China, examining the direct effect of childhood obesity on precocious puberty; II) exploring the relationship between prepuberty obesity and its risk genes and precocious puberty; III) determining the relationship between prepuberty obesity and its risk genes and hormone levels among children with precocious puberty; and IV) analyzing the interaction effect between genes and environmental factors on precocious puberty.

## Methods

### Study design, setting, and participants

#### Study design and sampling

This study is a prospective multi-center cohort study sponsored by the Shanghai Children’s Medical Center affiliated to the School of Medicine, Shanghai Jiao Tong University and performed in three cities in southern, central, and northern China, namely, Zhongshan, Guangdong Province, Qufu, Shandong Province, and Dalian, Liaoning Province. According to socioeconomic level and geographical distribution, the districts of Zhongshan, Qufu, and Dalian are stratified into urban districts in the central area and suburban districts in suburban areas. Using a stratified cluster random sampling method, districts and schools will be selected according to geographical location and school size by a random number generator. Grade 1, 2, and 3 children (6 to 10 years old) from primary schools will be included in this study. However, participants with overweight or obesity with a history of hormone treatment in the past 6 months as reported by parents in the questionnaire will be excluded.

We will carry out a nested case-control study, which will be nested in this cohort study. Participants in case-control study will be enrolled in four groups: children with normal weight and normal puberty, with normal weight and precocious puberty, with overweight/obesity and normal puberty, and with overweight/obesity and precocious puberty. And they will be measured body composition, collected venous blood, and stool at baseline, and collected urine samples at baseline and follow-up periods.

#### Sample size

Based on the formula for calculating the sample size of a cohort study at one-side significance level: [22].

$$N={\left[\frac{u_{\alpha}\sqrt{2\overline{p}\left(1-\overline{p}\right)+}{u}_{\beta}\sqrt{p_1\left(1-{p}_1\right)+{p}_2\left(1-{p}_2\right)}}{p_1-{p}_2}\right]}^2$$

According to the previous studies, the prevalence of precocious puberty was 4.85% (*P*_2_) and 8.43% (*P*_1_) of children aged 7–10 years with normal weight and overweight, respectively [12, 23]. A type I error (*α*) of 0.05, u_0.05_ = 1.96;, β = 0.10, u_0.10_ = 1.28; and a loss of follow up rate of 15%, a total of 4152 children will be required in each center. Finally, inverse-variance weighting (IVW) methods will be used to adjust the sample size.

Based on the SNP gene differences between different groups reported in the literature, Quanto 1.2.4 software (John Morrison, W. James Gauderman 2009, University of Southern California) was used to estimate sample sizes for each group. According to the previous pre-experimental results of obesity study showed that the allele frequency was 0.30 and the minimum allele frequency (MAF) was 0.10 using Additive Genetic Model (ADD), the type I error (α) of 0.05, the actual Power is 0.80, we need average of 800 participants per group and 3200 cases for four groups. Cases among four groups will be controlled by sex and age.

#### Study procedure

**Fig. 1:**
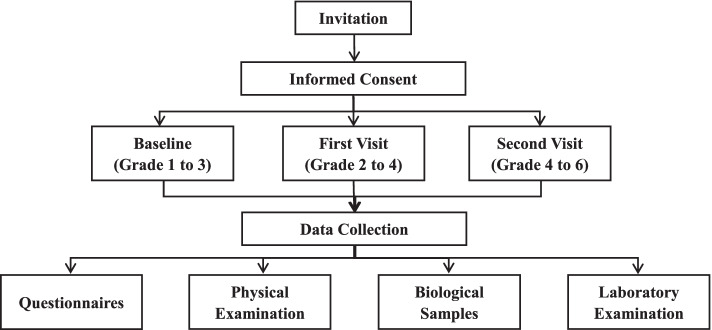
Flow chart of the prospective cohort study

Participants will accept anthropometric measuring by clinicians from the three collaborating hospitals, including Bo Ai Hospital of Zhongshan, People’s Hospital of Qu Fu, and Da Lian Children’s Hospital. The cohort study was scheduled to begin in October, 2019, and the first and secondary follow-up visits will be conducted one and two years later, respectively. A flowchart of the study design was shown in Fig. 1, and the investigation contents required at baseline, first visit, and second visit were shown in Table 1.

**Table 1:** The investigation content required at baseline, follow-up of the prospective cohort study

Items	Baseline	First visit	Second visit
**Children’s health and family environment questionnaire (all participants)**			
Lifestyle	√	√	√
Family environment	√	√	√
Parental information	√	√	√
Early life information	√	√	√
**Physical examination (all participants)**			
Anthropometric measures	√	√	√
Sexual development	√	√	√
**Biological samples (participants selected for case-control study)**			
Blood	√		
Urine	√	√	√
Feces	√		
**Laboratory examination (participants selected for case-control study)**			
Endogenous hormones	√	√	√
Genes	√		
EEDs	√		
Gut microbiota	√		

*EEDs* Environment endocrine disruptors

### Data collection

#### Genotyping and SNP selection

Due to differences in gene polymorphism distribution in different ethnic groups, we screened ten candidate genes and selected one or two representative single-nucleotide polymorphisms (SNPs) each gene on the basis of the Hans according to GWAS database (www.genome.gov/gwastudies) and the related literatures, the candidate genes and SNPs were shown in Table 2.

**Table 2:** The characteristics of candidate gene associated with obesity and precocious puberty

Gene	Chr	SNP1	SNP2
*ADCY3*	2p23.3	rs6752378	rs713586
*BDNF*	11p14.1	rs4923461	rs7481311
*FTO*	16q12.2	rs1121980	rs1558902
*KCTD15*	19q12.1	rs29941	rs287103
*LEP*	7q32.1	rs104894023	rs724159998
*NEGR1*	1p31.1	rs2568958	rs2815752
*SEC16B*	1q25.2	rs10913469	rs543874
*TMEM18*	2p25.3	rs4854344	rs2867125
*TNNI3K*	1p31.1	rs1040070	rs1514175
*MC4R*	18q22	rs663129	rs12970134

#### Physical examination

Anthropometric data, including weight (kg), height (cm), and waist circumference (cm), will be measured for each child standing without shoes and lightly clothed using standard techniques by one observer at baseline and the first and second visit. Waist circumference will be measured midway between the lower edge of the rib and the upper edge of the ilium. All of the measurement indexes will be measured twice and averaged. Overweight and obesity are the main predictors of interest and will be defined by BMI value, which is calculated as weight (kg) divided by height squared (m^2^). Participants will be defined as normal weight, overweight, or obese if their BMI index is between 18.5 and 25 kg/m^2^, greater than 25 kg/m^2^, or greater than 30 kg/m^2^, respectively, according to the age- and sex-specific cutoff standard for 2- to 18-year-old children proposed by the International Obesity Task Force (IOTF) [24, 25]. Children with overweight or obesity and some participants with normal weight (matched for sex and age with children with obesity and overweight) will be recruited to analyze their body composition, including height, weight, fat-free mass, body fat mass, body fat rate, body water, muscle mass, basal metabolic rate, total energy consumption, protein, inorganic salt, and impedance.

#### Sexual development assessment

The primary outcome is the difference in incidence of precocious puberty between normal-weight and obese children.

Secondary sexual characteristics assessment for boys and girls will be conducted by male and female professional pediatric endocrinologists or pediatric care physicians separately in different rooms to protect the children’s privacy. Breast development will be evaluated by visual inspection and palpation. Girls with overweight or obesity will be subjected to breast ultrasound to evaluate their nodules. In boys, testicular volume (TV) will be determined by palpation and an orchidometer. According to Marshall and Tanner [26, 27], breast and pubic hair development are divided into 5 stages (stages I to V). Precocious puberty will be diagnosed according to the Tanner stage: onset of breast development stage II (B_2_) or pubic hair development stage II (PH_2_) or above before 8 years or menarche before 10 years of age in girls, and PH_2_ or testicular enlargement (TV ≥ 4 ml) before 9 years of age in boys [28–30].

The presence of early puberty will be defined as children reaching a certain stage of puberty earlier than the median age of that stage [31]. According to previous studies, the median age of puberty onset (girls: breast or pubic hair development; boys: pubic hair or testicular volume development) of Chinese adolescents will be referenced [32, 33], and the onset of menarche before 12 years of age in girls and of voice breaking before 13 years of age in boys will also be defined as early puberty [34].

We separately evaluated the tanner stage for each breast among obese girls by using portable ultrasound (US), and chose the larger one as evaluation result. Tanner II by US is characterized as the presence of a hyperechoic retro-areolar nodule with a central star-shaped or linear hypoechoic area, this stage is assumed to mark the start of breast development [35].

#### Biospecimen collection and laboratory examination

Children with overweight or obesity and some of the participants with normal weight (matched for sex and age) will be recruited to collect venous blood at baseline; urine samples will be collected at baseline and 1-year follow-up. A total of 2 ml and 1 ml venous blood will be collected in coagulation and anticoagulation collection tubes, respectively. These participants will receive a vacuum stool collection tube and 15-ml urine tube 1 day before physical inspection, and they will be asked to collect stool and urine samples in the morning and take them to the researchers at school on the physical inspection day. All of the biological sample will be transferred with an ice bag to a local collaborating hospital within 2 hours after collection. Blood samples in coagulation collection tubes will be centrifuged to extract serum. Then, all biospecimens will be transferred with dry ice to the Biobank of Shanghai Children’s Medical Center and stored at − 80 °C.

Part of the serum sample will be used to test follicle-stimulating hormone (FSH), luteinizing hormone (LH), and estrogens (E2) in girls and testosterone (T) in boys; the residual serum will be used to test leptin, kisspeptin, and ghrelin levels. A whole-blood sample will be used to analyze the pleiotropic genes of precocious puberty and obesity.

Simultaneous quantification of 64 steroids, including EED in pediatric plasma and urine by LC–MS/MS. Using the SNaPshot technology of ultra-high throughput SNP typing to detect genetic markers, thousands of SNP loci can be detected in each reaction. ABI3730XL sequencer was used for simultaneous detection of several samples.

#### Children’s health and family environment questionnaire

i. Basic information of children, including age, sex, birth status, siblings, adoption, and physical and sexual development.
ii. Basic Information of Children’s Parents and Family Environment, including parents’ educational level, occupation, weight status and marital status; and family annual income, and address.
iii. A sleep pattern questionnaire, including wake-up time, bedtime, and daytime napping time on workdays and weekdays and chronotype (morning, intermediate, or evening type), is used to document the sleep/wake patterns of children in the past week.
iv. The dietary habit questionnaire, which consists of food consumption frequency, frequency of consuming plastic or tin packaged products, and frequency of consuming take-out food during the past week [36, 37].
v. Physical activity habits are assessed using the Physical Activity Questionnaire to record the days and times of high-, moderate-, and low-intensity activities of children during the past week [38].
vi. Items from the Pubertal Development Scale (PDS) are also adapted to assess puberty development, which is indicated by 4 items (growth spurt, pubic hair, skin changes, and breast development) in girls and by 5 items (growth spurt, pubic hair, skin changes, voice breaking, and facial hair) in boys. For each, the parent rates his or her child’s development as “has not started yet,” “has barely started,” “has definitely started,” “seems complete,” or “unclear.” For each adolescent, the PDS score is the mean of the sum of items [39].

### Data analysis

Categorical variables will be reported as the number and percentage and analyzed by a chi-square test to compare the distribution between groups. The *t*-test, analysis of variance, and Wilcoxon rank-sum test will be used to compare the differences in distribution of continuous variables, which will be described by mean ± standard deviation (*SD*) for normal data or median with interquartile range for non-normal data.

Based on the discovered pleiotropic genes and obesity-related risk loci (SNPS), we will examine the distribution of gene polymorphisms (SNPS). For obesity-related SNPs of these genes, Effect size Weighting was carried out according to the method reported by Speliotes EK et al. [40], and then genetic risk scores (GRS) will been calculated using the method established in our previous study [41].That is, the number of effective Allele genes (Allele) was calculated, then the weighted value was multiplied, and finally the combined GRS of SNPs of all candidate genes in this study was accumulated.

The effects of exposure factors on outcomes will be evaluated after adjusting confounding factors using logistic regression or multiple linear regression. To analyze whether there is a linear trend of GRS among four groups: children with normal weight and normal puberty, with normal weight and precocious puberty, with overweight/obesity and normal puberty, and with overweight/obesity and precocious puberty, the Cochran–Mantel–Haenszel Trend Test will be used. After 3 years’ follow-up, a causal relationship model between exposure factors and outcomes will be established by using Mendelian randomization (MR) and Egger’s regression models and assessed by sensitivity analysis. Statistical analyses will be performed by using IBM SPSS, STATA and R statistical packages. and the level of significance will be set at *p* < 0.05.

## Discussion

In this protocol, we described the study design, data collection methods, and analysis of a prospective cohort study in a population-based primary school children. According to the geographical distribution, we chose three cities in northern, central, and southern China and did our best to carry out this study to ensure the representativeness of the population. Students in grades 1 to 3 in randomly selected primary schools are expected to represent local school-age children.

By collecting information on factors such as children’s family environment, lifestyle, and genes, this study will analyze the effect of these factors on children’s physical development from the perspectives of heredity and environment. As a longitudinal study, this study will start earlier in development than previous studies to capture the impact of early childhood experiences on puberty development. Puberty is defined through visual and palpation inspection by a clinical doctor combined with ultrasonic diagnostics; previous studies have depended on self-reports or clinical palpation by non-professional volunteers, which are less sensitive and precise measurements, especially for breast development assessment among obese girls, which may lead to overestimating the precocious puberty detection rate among obese girls, since it is difficult to distinguish adipose tissue and breast tissue, so we will use ultrasonic examination in this study. Although the relationship between obesity and the sexual development of children has been confirmed in both genders by many earlier studies, due to the racial differences in the influence of obesity on sexual precocity, the relationship between obesity and early development is controversial among boys. Through this prospective cohort study, we will further investigate the relationship between obesity and development among Chinese mainland children.

To expand the previous research on puberty development, this study starts from the gene pleiotropy of obesity and takes the lead in studying the influence of the genetic polymorphism of obesity on precocious puberty. Although studies on genetic polymorphism of obesity or precocious puberty in girls have been performed previously, there has been no report on the combination of the two phenotypes, which is expected to reveal the genetic mechanism of the association between childhood obesity and precocious puberty.

At the same time, this study adopts several new analysis techniques, such as Mendelian randomization and Egger’s test, to verify potential pleiotropic genes and further identify the mediating role of obesity in the relationship between pleiotropic genes and precocity. There is a lack of understanding of the direction and extent of the effect of life behaviors and habits on the internal rhythm and timing of puberty and how external factors interact with genetics to influence the indicators of adolescent development, such as secondary sexual characteristics development and sex hormone levels. Through these analysis methods, we can understand the direct and indirect effects of these influence factors on child growth and development.

This study has several limitations that should be considered. First, information on the diets and physical activity of children will be collected through parent reports, which may lead to bias due to a lack of information on children’ behaviors at school. Second, detection of the number of genes and SNP is limited by research funding, and there can be differences in gene polymorphism distribution in different ethnic groups; therefore, we are basing our analysis on Han people according to the GWAS directory database (www.genome.gov/gwastudies) and literature reports on potential candidate genes to screen pleiotropic genes related to obesity and precocious puberty. However, only 2 SNPs per candidate gene will be tested in this study. Despite these limitations, China is a country of great ethnic and genetic diversity. Up to now, there have been few prospective cohort studies on obesity and puberty development in China. Through this multicenter prospective population-based cohort study, we expect to find that obesity and the external environment indeed have an impact on puberty development in Chinese children. It is possible to develop directional intervention measures on the basis of the established evidence for youth at risk in families, communities, or schools to improve the adolescent health.

### Study status

Enrollment of prospective longitudinal study participants had begun in October 2019. However, due to the COVID-19 epidemic, our investigation in different city cannot carried out simultaneously. Currently (as of April 2022), data collection of baseline survey in Zhongshan, Guangdong province and Qufu, Shandong province were done in December 2020, and the first year follow-up in two of above cities were finished in January 2022. Data collection and the analysis of the results is ongoing. The baseline survey in Dalian, Liaoning province is scheduled to begin in December 2022. The baseline characters of this survey in Zhongshan, Guangdong province and Qufu, Shandong province were summarized in Table 3.

**Table 3:** The basic characteristics of the subjects in Qufu and Zhongshan

Category	Girls	Boys	Total
	3993 (%)	4630 (%)	8623 (%)
**Age (years)**			
6 ≤ age<8	2365 (59.23)	2821 (60.93)	5186 (60.14)
8 ≤ age<11	1628 (40.77)	1809 (39.07)	3437 (39.86)
**Districts**			
Urban	3151 (78.91)	3518 (75.98)	6669 (77.34)
Suburban	842 (21.09)	1112 (24.02)	1954 (22.66)
**Weight status**			
Thinness	720 (18.04)	629 (13.59)	1349 (15.64)
Normal weight	2594 (64.96)	2801 (60.50)	5395 (62.57)
Overweight or obesity	679 (17.00)	1200 (25.91)	1879 (21.79)

## Data Availability

The datasets used and/or analyzed during the current study are available from the corresponding author on reasonable request.

## Abbreviations

BMI: Body mass index
TV: Testicular volume
B: Breast
PH: Pubic hair
FSH: Follicle-stimulating hormone
LH: Luteinizing hormone
E _2_: Estrogens
T: Testosterone
EED: Environmental endocrine disruptors
SD: Standard deviation
PDS: Pubertal Development Scale
GRS: Genetic risk score
MR: Mendelian randomization
SNP: Single-nucleotide polymorphism
